# Preoperative serum CA19-9 should be routinely measured in the colorectal patients with preoperative normal serum CEA: a multicenter retrospective cohort study

**DOI:** 10.1186/s12885-022-10051-2

**Published:** 2022-09-08

**Authors:** Zhenhui Li, Haibin Zhu, Xiaolin Pang, Yun Mao, Xiaoping Yi, Chunxia Li, Ming Lei, Xianshuo Cheng, Lei Liang, Jiamei Wu, Yingying Ding, Jun Yang, Yingshi Sun, Tao Zhang, Dingyun You, Zaiyi Liu

**Affiliations:** 1 Department of Radiology, Guangdong Provincial People’s Hospital, Guangdong Academy of Medical Sciences, Guangzhou, 510080 China; 2 Guangdong Cardiovascular Institute, Guangzhou, 510080 China; 3 Guangdong Provincial Key Laboratory of Artificial Intelligence in Medical Image Analysis and Application, Guangdong Provincial People’s Hospital, Guangdong Academy of Medical Sciences, Guangzhou, 510080 China; 4 Department of Radiology, Third Affiliated Hospital of Kunming Medical University, Yunnan Cancer Hospital, Yunnan Cancer Center, Kunming, 650118 China; 5 Key Laboratory of Carcinogenesis and Translational Research (Ministry of Education/Beijing), Department of Radiology, Peking University Cancer Hospital & Institute, Beijing, 100142 China; 6 Department of Radiotherapy, Sixth Affiliated Hospital of Sun Yat-Sen University, Guangzhou, 510655 China; 7 Department of Radiology, the First Affiliated Hospital of Chongqing Medical University, Chongqing, 400016 China; 8 Department of Radiology, Xiangya Hospital, Central South University, Changsha, 410008 China; 9 Department of Biostatistics, School of Public Health, Cheeloo College of Medicine, Shandong University, Jinan, 250012 Shandong China; 10 Department of Clinical Laboratory Medicine, Third Affiliated Hospital of Kunming Medical University, Yunnan Cancer Hospital, Yunnan Cancer Center, Kunming, 650118 China; 11 Department of Colorectal Surgery, Third Affiliated Hospital of Kunming Medical University, Yunnan Cancer Hospital, Yunnan Cancer Center, Kunming, 650118 China; 12 Department of Oncology, the First Affiliated Hospital of Kunming Medical University, Kunming, 650032 China; 13 School of Public Health, Kunming Medical University, Kunming, 650500 Yunnan China

**Keywords:** Colorectal cancer, CA19-9, CEA, Interaction

## Abstract

**Objective:**

Whether preoperative serum carbohydrate antigen 19–9 (CA19-9) is an independent prognostic factor and there are interactions of serum CA19-9 with carcinoembryonic antigen (CEA) on the risk of recurrence in colorectal cancer (CRC) patients are still not clarified.

**Methods:**

Consecutive patients with CRC who underwent curative resection for stage II-III colorectal adenocarcinoma at five hospitals were collected. Based on Cox models, associations of preoperative CA19-9 with recurrence-free survival (RFS) and overall survival (OS) were evaluated in patients with or without elevated CEA, and interactions between CEA and CA19-9 were also calculated. Restricted cubic spline (RCS) curves were used to evaluate the associations between preoperative CA19-9 and CRC outcomes on a continuous scale.

**Results:**

A total of 5048 patients (3029 [60.0%] men; median [interquartile range, IQR] age, 61.0 [51.0, 68.0] years; median [IQR] follow-up duration 46.8 [36.5–62.4] months) were included. The risk of recurrence increased with the elevated level of preoperative CA19-9, with the slope steeper in patients with normal CEA than those with elevated CEA. Worse RFS was observed for elevated preoperative CA19-9 (> 37 U/mL) (*n* = 738) versus normal preoperative CA19-9 (≤ 37 U/mL) (*n* = 4310) (3-year RFS rate: 59.4% versus 78.0%; unadjusted hazard ratio [HR]: 2.02; 95% confidence interval [CI]:1.79 to 2.28), and significant interaction was found between CA19-9 and CEA (P for interaction = 0.001). Increased risk and interaction with CEA were also observed for OS. In the Cox multivariable analysis, elevated CA19-9 was associated with shorter RFS and OS regardless of preoperative CEA level, even after adjustment for other prognostic factors (HR: 2.08, 95% CI:1.75 to 2.47; HR: 2.25, 95% CI:1.80 to 2.81). Subgroup analyses and sensitivity analyses yielded largely similar results. These associations were maintained in patients with stage II disease (*n* = 2724).

**Conclusions:**

Preoperative CA19-9 is an independent prognostic factor in CRC patients. Preoperative CA19-9 can be clinically used as a routine biomarker for CRC patients, especially with preoperative normal serum CEA.

**Supplementary Information:**

The online version contains supplementary material available at 10.1186/s12885-022-10051-2.

## Background

Carbohydrate antigen 19–9 (CA19-9) is a commonly used serum biomarker for early diagnosis, treatment response, recurrence monitoring, and prognosis in pancreatic cancer, as well as several other cancers of the gastrointestinal tract [1–6]. Despite of its wide use in clinical practice, the value of CA19-9 in prognosis prediction in patients with colorectal cancer (CRC) is not completely understood [7–9]. CA19-9 has been associated with prognosis in CRC patients independent of existing prognostic factors including T stage, N stage, carcinoembryonic antigen (CEA) in some studies [10–21], however, not in other studies [22–26]. A recent meta-analysis indicated that patients with elevated CA19-9 have shorter overall survival (hazard ratios [HR]: 1.58, 95% confidence interval [CI]: 1.36–1.83), disease-free survival (HR: 1.71, 95% CI: 1.38–2.13), and recurrence-free survival (RFS) (HR: 1.43, 95% CI: 1.11–1.83), but only 4 out of the 17 studies included in the meta-analysis had a sample size > 400. Hence, there is insufficient data, especially international multicenter data, to definite the prognostic value of preoperative CA19-9 in CRC to date.

The role of CA19-9 in addition to the guideline-recommended CEA in the prognosis prediction of postoperative CRC is of clinical concern. Several studies suggested that it is poorer survival in patients with elevated level of both preoperative CA19-9 and CEA level vs. in patients with elevated CA19-9 or CEA alone [19, 27], and they believed that the combination of preoperative CEA and CA19-9 were helpful for predicting prognosis of CRC after radical resection. Stiksma et al. found that patients with elevated preoperative CA19-9 levels had worse 5-year survival than patients with elevated preoperative CEA levels, and suggested that CA19-9 be used to monitor disease progression in CRC patients without elevated CEA [17]. Lin et al. concluded that elevated CA19-9 predicts poor survival only in patients with normal preoperative CEA level [16]. These results suggested that the prognostic impact of CA19-9 may be dependent on preoperative CEA level in CRC [16–19, 27]. Therefore, we hypothesize that there is an interaction between CEA and CA19-9 on the prognosis of CRC and design a multicenter cohort to explore it.

We conducted a large-scale multicenter retrospective cohort study to verify whether preoperative CA19-9 is an independent prognostic factor in stage II-III CRC patients and further whether the prognostic impact of CA19-9 is dependent on CEA status.

## Patients and methods

### Patients

**Fig. 1:**
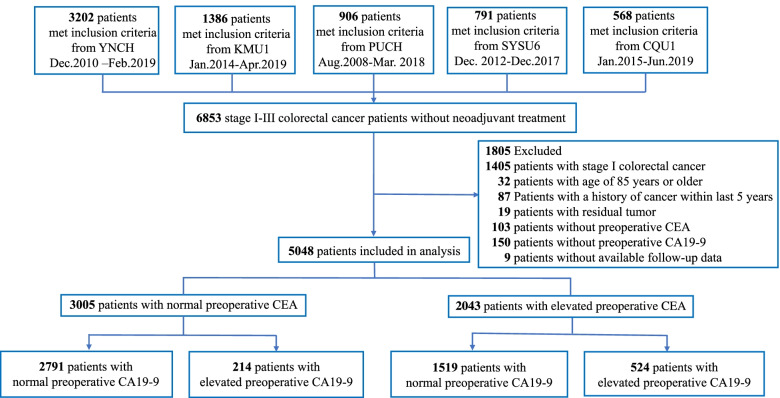
Flow Chart of cohort selection. CQU1, the First Affiliated Hospital of Chongqing Medical University; KMU1, the First Affiliated Hospital of Kunming Medical University; PUCH, Peking University Cancer Hospital & Institute; SYSU6, the Sixth Affiliated Hospital of Sun Yat-sen University; YNCH, Yunnan Cancer Hospital

The data analysis included consecutive patients with stage II-III receiving radical resection at the following tertiary hospitals: from August 2008 to March 2018 at Peking University Cancer Hospital & Institute, from December 2010 to February 2019 at Yunnan Cancer Hospital, from December 2012 to December 2017 at the Sixth Affiliated Hospital of Sun Yat-sen University, from January 2015 to June 2019 at the First Affiliated Hospital of Chongqing Medical University, and from January 2014 to April 2019 at the First Affiliated Hospital of Kunming Medical University. Patients receiving neoadjuvant treatment were excluded from the analysis. The study flowchart, including the inclusion and exclusion criteria, is shown in Fig. 1.

Extracted variables included age, sex, serum CA19-9, serum CEA, primary sites (colon or rectum), surgical approach (open resection or laparoscopic resection), tumor differentiations, T-stage, N-stage, lymph node yield (≥ 12 or < 12), mucinous (colloid) type (yes or no), the presence of lymphovascular/ perineural invasion (yes or no), microsatellite instability (MSI) status (yes or no), and the adjuvant chemotherapies (yes or no).

### Serum CA19-9 determination

Preoperative CA19-9 level closest to the time of surgery within four weeks before surgery was used in the analysis. Serum CA19-9 was measured with a chemiluminescence immunoassay using the COBAS e601 immunoassay analyzer (Roche Diagnostics, Tokyo, Japan) at Peking University Cancer Hospital & Institute, COBAS e602 immunoassay analyzer (Roche Diagnostics, Tokyo, Japan) at Yunnan Cancer Hospital, Alinity i immunoassay analyzer (Abbott Diagnostics, Chicago, USA) at the Sixth Affiliated Hospital of Sun Yat-sen University, COBAS e602 immunoassay analyzer (Roche Diagnostics, Tokyo, Japan) at the First Affiliated Hospital of Chongqing Medical University, and COBAS e601 immunoassay analyzer (Roche Diagnostics, Tokyo, Japan) at the First Affiliated Hospital of Kunming Medical University. CA19-9 at > 37 U/mL was considered elevated.

### Surveillance protocol and outcome

Serum CEA was examined at 3–6 months intervals during the first 2 years after surgery and every 6 months thereafter. Contrast-enhanced computed tomography of the chest, abdomen, and pelvis was performed at a minimum of every 12 months for at least three years. Colonoscopy was performed one year after surgery and every 2–5 years thereafter unless warranted otherwise (e.g., identification of advanced adenomas). Recurrence-free survival (RFS), as assessed by biopsy or imaging, was measured from the date of surgery to the verified first recurrence (local or distant) or death from any cause and was censored at the last follow-up (31 August 2021) [28]. Additional outcome of interest was overall survival (OS), namely the time from surgery to death due to any cause.

### Statistical analysis

This study was conducted in compliance with the REMARK guideline [29] and STROBE guideline [30].Continuous variables are shown as mean values ± standard deviations (SD) (normal distribution) or median (quartile) (skewed distribution). Categorical variables are shown as frequency or percentage. The association of CA19-9 with clinicopathological characteristics was assessed using Mann–Whitney U test or Student T-test according to normality assumption for continuous variables and χ^2^ statistics for categorical variables.

The association between CA19-9 and all outcome measures were evaluated on a continuous scale with restricted cubic spline (RCS) curves based on Cox proportional hazards models [31]. RCS presents a smooth curve of continuous variables over the entire value range, and has been widely used to describe the nonlinear relationship between continuous independent variables and survival. Its essence is a piecewise cubic polynomial fitted by choosing the number and position of knots [32]. The number of knots determines the shape of the curve and has a greater impact on the RCS function, which is decided by AIC. To choose an appropriate number of knots, we traversed 3–10 knots, and finally the RCS curve with 4 knots was determined. The location of the knots has little effect on the fitting of the RCS function, which is generally placed at fixed quantiles of continuous predictor’s marginal distribution. For knots locations, Harrell et al. gave recommended equally spaced quantiles [32]. In conclusion, the spline was defined using four knots at the 5th, 35th, 65th and 95th percentiles. Logarithms of preoperative CA19-9 was used for RCS due to non-normality, and the threshold was defined as the clinical cut-off point of preoperative CA19-9 (37 U/ml). The 95% CI was derived by bootstrap resampling. RCS analysis was conducted using package “rms” (version 5.1–4) in R (version 3.6.3).

RFS and OS were analyzed using the Kaplan–Meier analysis followed a log-rank test. We calculated the follow-up the reverse Kaplan–Meier estimation. The association between CA19-9 and RFS/OS was analyzed in the entire cohort as well as separately in patients with normal vs. elevated CEA. Results are shown as HR with 95% CI. A total of four models were used: no adjustment (model 1); adjustment for sex and age (model 2); adjustment for sex, age, primary site, surgical approach, pathology stage, lymph node yield, tumor differentiation, mucinous (colloid) type, lymphovascular invasion / perineural invasion, adjuvant chemotherapy (model 3); adjustment for factors in model 3 plus MSI status (model 4).

Robustness of the risk estimates was examined using a frailty model analysis that introduces random effects in the model to account for heterogeneity across different centers [33] and a repeat analysis using 74 U/mL cutoff (rather than 37 U/mL) for CA19-9 [34].

Subgroup analyses were performed based on, sex, age, primary site, surgical approach, cancer stage, tumor differentiation, lymph node yield, adjuvant chemotherapy, and center, with tests for interaction by the Cox regression model.

All analyses all two-sided and conducted using the R software (version 3.6.3; http://www.R-project.org). Statistical significance set at a *P*-value < 0.05.

## Results

### Patient characteristics

A total of 6853 patients were screened. 1805 (26.3%) were excluded from the analysis for the following reasons: stage I (*n* = 1405), 85 years of age or older (*n* = 32), a history of cancer within 5 years prior to surgery (*n* = 87), residual tumor after surgery (*n* = 19), no preoperative CEA data (*n* = 103), no preoperative CA19-9 data (*n* = 150), and loss to follow-up (*n* = 9) (Fig. 1). The final analysis included 5048 patients: 738 (14.6%) with elevated CA19-9 and 4310 (85.4%) with normal CA19-9. The median (IQR) CA19-9 and CEA levels were 11.9 [7.3, 23.4] U/ml and 3.8 [2.1, 9.4] ng/mL, respectively. Within the median follow-up of 46.8 months (interquartile range [IQR]: 36.5–62.4; range 0.8–129.6 months), 1488 patients (29.5%) had recurrence, and 898 patients (17.8%) died. Baseline characteristics of the entire cohort, as well as in patients with elevated vs. normal CA19-9 are shown in Table 1. And baseline characteristics of the five cohorts of patients are listed in Table S1.

**Table 1:** Baseline characteristics

Variable	Total (*n* = 5048)	Preoperative CA19-9 group		*P* value
		**Normal CA19-9 (n = 4310)**	**Elevated CA19-9 (n = 738)**	
Hospital, n (%)				< 0.001
YNCH	2170 (43.0)	1843 (42.8)	327 (44.3)	
KYU1	1111 (22.0)	986 (22.9)	125 (16.9)	
PUCH	604 (12.0)	541 (12.6)	63 (8.5)	
SYSU6	683 (13.5)	545 (12.6)	138 (18.7)	
CQU1	480 (9.5)	395 (9.2)	85 (11.5)	
Male, n (%)	3029 (60.0)	2629 (61.0)	400 (54.2)	0.001
Age^a^	61.0 [51.0, 68.0]	61.0 [51.0, 68.0]	62.0 [51.0, 69.0]	0.114
Preoperative CEA, ng/ml ^a^	3.8 [2.1, 9.4]	3.5 [2.0, 7.3]	10.6 [4.3, 28.1]	< 0.001
Preoperative CEA group, n (%)				< 0.001
≥ 5 ng/ml	2043 (40.5)	1519 (35.2)	524 (71.0)	
< 5 ng/ml	3005 (59.5)	2791 (64.8)	214 (29.0)	
Preoperative CA19-9, U/ml ^a^	11.9 [7.3, 23.4]	11.4 [6.4, 16.8]	69.7 [48.3, 143.5]	< 0.001
Primary site, n (%)				< 0.001
Colon	2659 (52.7)	2216 (51.4)	443 (60.0)	
Rectum	2389 (47.3)	2094 (48.6)	295 (40.0)	
Surgical approach, n (%)				0.079
Laparoscopic resection	3010 (59.6)	2596 (60.2)	414 (56.1)	
Open resection	2035 (40.3)	1711 (39.7)	324 (43.9)	
Unknown	3 (0.1)	3 (0.1)	0 (0.0)	
AJCC 8th ed. Stage, n (%)				
II	2724 (54.0)	2403 (55.8)	321 (43.5)	< 0.001
III	2324 (46.0)	1907 (44.2)	417 (56.5)	
Lymph node yield, n (%)				0.216
≥ 12	3883 (76.9)	3305 (76.7)	578 (78.3)	
< 12	1163 (23.0)	1004 (23.3)	159 (21.5)	
Unknown	2 (0.0)	1 (0.0)	1 (0.1)	
Tumor differentiation, n (%)				0.002
Well-moderate	3533 (70.0)	3052 (70.8)	481 (65.2)	
Poor-undifferentiated	1040 (20.6)	853 (19.8)	187 (25.3)	
Unknown	475 (9.4)	405 (9.4)	70 (9.5)	
Lymphovascular / Perineural invasion, n (%)				0.020
Yes	1168 (23.1)	969 (22.5)	199 (27.0)	
No	3759 (74.5)	3240 (75.2)	519 (70.3)	
Unknown	121 (2.4)	101 (2.3)	20 (2.7)	
MSI, n (%)				0.272
Yes	886 (17.6)	751 (17.4)	135 (18.3)	
No	2354 (46.6)	2030 (47.1)	324 (43.9)	
Unknown	1808 (35.8)	1529 (35.5)	279 (37.8)	
Adjuvant chemotherapy, n (%)				0.261
Yes	3576 (70.8)	3035 (70.4)	541 (73.3)	
No	1471 (29.1)	1274 (29.6)	197 (26.7)	
Unknown	1 (0.0)	1 (0.0)	0 (0.0)	
Mucinous (colloid) type, n (%)				< 0.001
Yes	395 (7.8)	303 (7.0)	92 (12.5)	
No	4648 (92.1)	4002 (92.9)	646 (87.5)	
Unknown	475 (9.4)	405 (9.4)	70 (9.5)	

Note: ^a^ Data is median [IQR]

*CA 19–9* carbohydrate antigen 19–9, *CEA* carcinoembryonic antigen, *MSI* microsatellite instability, *CQU1* the First Affiliated Hospital of Chongqing Medical University, *KMU1* the First Affiliated Hospital of Kunming Medical University, *PUCH* Peking University Cancer Hospital & Institute, *SYSU6* the Sixth Affiliated Hospital of Sun Yat-sen University, *YNCH* Yunnan Cancer Hospital

### Association between CA19-9 and outcome and interactions with CEA

**Fig. 2:**
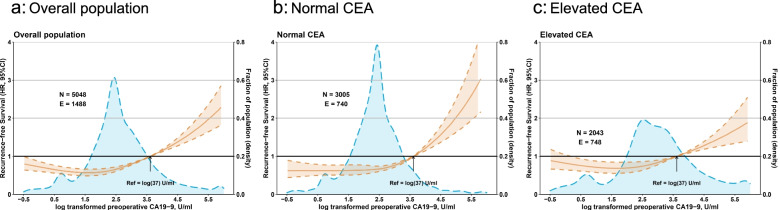
Association between preoperative CA19-9 status and recurrence-free survival. (a) overall population. (b) patients with normal preoperative CEA. (c) patients with elevated preoperative CEA. Solid yellow lines are unadjusted hazard ratios, with dashed yellow lines showing 95% confidence intervals derived from restricted cubic spline regressions. Reference lines for no association are indicated by the solid bold lines at a hazard ratio (HR) of 1.0. Dashed blue curves show the fraction of the population with different levels of preoperative CA19-9. Arrows indicate the concentration of preoperative CA19-9 with HR of 1.0. CA19-9, carbohydrate antigen 19–9; CEA, carcinoembryonic antigen; CI, confidence interval; E, number of events; HR, hazard ratio; N, number of patients

The risk of recurrence was relatively stable when preoperative CA19-9 was lower than 37 U/ml, and began to increase significantly after preoperative CA19-9 exceeded 37 U/ml. (Fig. 2a). Such an association between levels of preoperative CA19-9 on a continuous scale and risk of recurrence was evident in the analysis that included patients with normal preoperative CEA (≥ 5 ng/ml) (Fig. 2b) as well as in the analysis that included patients with elevated preoperative CEA (< 5 ng/ml) only (Fig. 2c), and the slope of increase was steeper in patients with normal CEA than those with elevated CEA. Interaction between CA19-9 and CEA on RFS was significant (*P* < 0.001). Similar associations between CA19-9 status and OS were observed (Supplementary Figure S1).

**Fig. 3:**
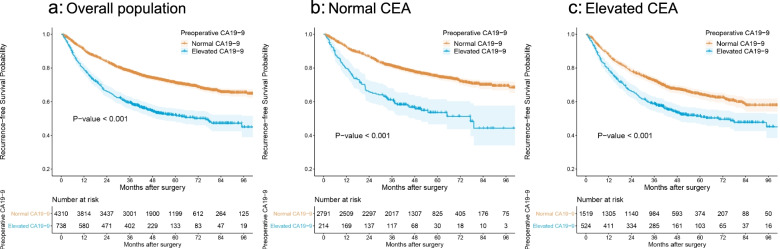
Kaplan‐Meier curves for recurrence-free survival according to the preoperative CA19-9 group. (a) overall population. (b) patients with normal preoperative CEA. (c) patients with elevated preoperative CEA. CA19-9, carbohydrate antigen 19–9; CEA, carcinoembryonic antigen

The 3-year RFS was 59.4% (55.9%-63.1%) and 78.0% (76.8%-79.3%) in patients with elevated and normal preoperative CA19-9, respectively (unadjusted HR = 2.15, 95% CI: 1.88–2.45, log-rank *P* < 0.001) (Fig. 3a). The 5-year OS was 65.9% (62.1%-69.9%) and 82.3% (80.9%-83.6%) in patients with elevated and normal preoperative CA19-9, respectively (unadjusted HR = 2.36, 95% CI: 1.88–2.45, log-rank *P* < 0.001) (Supplementary Figure S2a). Elevated CA19-9 was associated with poor RFS (unadjusted HR: 2.02, 95% CI: 1.79–2.28, *P* < 0.001) and OS (unadjusted HR:2.28, 95% CI: 1.96–2.65, *P* < 0.001) in a univariable Cox model (model 1). The adjustment resulted in a slight attenuation of the risk estimates in the model 2, model 3 and model 4 (Table 2, Supplementary Tables S2 and S3).

**Table 2:** Cox proportional hazard regression analysis of preoperative CA19-9 on colorectal cancer outcomes

Outcome	Total		Normal CEA group		Elevated CEA group	
	**Hazard Ratio (95% CI)**	***P* Value**	**Hazard Ratio (95% CI)**	***P* Value**	**Hazard Ratio (95% CI)**	***P* Value**
**RFS**						
Model1	2.02 (1.79–2.28)	< 0.001	2.34 (1.89–2.90)	< 0.001	1.56 (1.34–1.82)	< 0.001
Model2	2.02 (1.79–2.28)	< 0.001	2.41 (1.94–2.99)	< 0.001	1.56 (1.34–1.82)	< 0.001
Model3	1.90 (1.67–2.16)	< 0.001	2.10 (1.66–2.66)	< 0.001	1.54 (1.30–1.81)	< 0.001
Model4	2.08 (1.75–2.47)	< 0.001	2.01 (1.47–2.74)	0.001	1.68 (1.35–2.08)	< 0.001
**OS**						
Model1	2.28 (1.96–2.65)	< 0.001	2.85 (2.18–3.72)	< 0.001	1.64 (1.36–1.98)	< 0.001
Model2	2.26 (1.95–2.63)	< 0.001	3.02 (2.31–3.95)	< 0.001	1.63 (1.35–1.97)	< 0.001
Model3	2.05 (1.74–2.42)	< 0.001	2.54 (1.89–3.42)	< 0.001	1.55 (1.27–1.90)	< 0.001
Model4	2.25 (1.80–2.81)	< 0.001	2.20 (1.44–3.35)	< 0.001	1.72 (1.30–2.28)	< 0.001

Note: *CA 19–9* carbohydrate antigen 19–9, *CEA* carcinoembryonic antigen, *OS* overall survival, *RFS* recurrence-free survival

Model 1 was unadjusted. Model 2 was adjusted for sex (female vs. male), age. Model 3 was adjusted for sex (female vs. male), age, primary site (rectum vs. colon), surgical approach (open resection vs. laparoscopic resection), pathology stage (III → II), lymph node yield (≥ 12 vs. < 12), tumor differentiation (poor-undifferentiated vs. moderate vs. well), mucinous (colloid) type (yes vs. no), lymphovascular invasion / perineural invasion (yes vs. no), adjuvant chemotherapy (yes vs. no). Model 4 was adjusted for sex (female vs. male), age, primary site (rectum vs. colon), surgical approach (open resection vs. laparoscopic resection), pathology stage (III → II), lymph node yield (≥ 12 vs. < 12), tumor differentiation (poor-undifferentiated vs. moderate vs. well), mucinous (colloid) type (yes vs. no), lymphovascular invasion / perineural invasion (yes vs. no), adjuvant chemotherapy (yes vs. no), microsatellite instability (yes vs. no)

In the analysis that included the patients with elevated preoperative CEA, the 3-year RFS was 58.7% (54.5%-63.1%) vs. 72.3% (70.1%-74.6%) in patients with elevated vs. normal preoperative CA19-9 (HR:1.56, 95% CI: 1.34–1.82, *P* < 0.001). In the analysis that included the patients with normal preoperative CEA, the 3-year RFS was 61.0% (54.8%-68.0%) vs. 81.0% (79.7%-82.6%) in patients with elevated vs. normal preoperative CA19-9 (HR: 2.34, 95% CI: 1.89–2.90, *P* < 0.001). There was a significant interaction between CA19-9 and CEA (P for interaction = 0.003; Fig. 3b and 3c, Supplementary Tables S4,S5, and S6). Similar interaction between CA19-9 and CEA was noted for OS (*P* for interaction = 0.001; Supplementary Tables S4, S7, and S8, Figure S2). The adjustment resulted in a slight attenuation of the HR estimates for RFS and OS, but the interaction remained despite of the adjustments with the exception for model 4. (Table 2, Supplementary Tables S4).

In multivariable analyses with adjustment, the HR on RFS was 1.65 (95% CI: 1.40–1.95, *P* < 0.001) in patients with elevated vs. normal preoperative CEA, and 2.00 (95% CI: 1.46–2.72, *P* < 0.001) in patients with elevated vs. normal preoperative CA19-9. The HR on RFS in patients with both elevated preoperative CEA and CA19-9 was 2.76 (95% CI: 2.24–3.39, *P* < 0.001) (Table 3 and Supplementary Figure S3a). Higher risk for OS was also evident in patients with both elevated preoperative CEA and CA19-9 (HR: 3.23, 95% CI: 2.46–4.24, *P* < 0.001) (Table 3 and Supplementary Figure S3b).

**Table 3:** Joint effect of preoperative CEA and CA19-9 on colorectal cancer outcomes

Outcome	Model 1		Model 2		Model 3		Model 4	
	**Hazard Ratio** **(95% CI)**	***P* Value**	**Hazard Ratio (95% CI)**	***P* Value**	**Hazard Ratio (95% CI)**	***P* Value**	**Hazard Ratio (95% CI)**	***P* Value**
**RFS**								
Normal CEA & normal CA19-9	Reference		Reference		Reference		Reference	
Normal CEA & elevated CA19-9	2.32 (1.87–2.88)	< 0.001	2.38 (1.92–2.96)	< 0.001	2.08 (1.65–2.62)	< 0.001	2.00 (1.46–2.72)	< 0.001
Elevated CEA & normal CA19-9	1.54 (1.37–1.73)	< 0.001	1.52 (1.35–1.71)	< 0.001	1.50 (1.32–1.70)	< 0.001	1.65 (1.40–1.95)	< 0.001
Elevated CEA & elevated CA19-9	2.41 (2.08–2.8)	< 0.001	2.38 (2.05–2.76)	< 0.001	2.31 (1.97–2.71)	< 0.001	2.76 (2.24–3.39)	< 0.001
**OS**								
Normal CEA & normal CA19-9	Reference		Reference		Reference		Reference	
Normal CEA & elevated CA19-9	2.85 (2.18–3.72)	< 0.001	3.02 (2.31–3.94)	< 0.001	2.52 (1.88–3.37)	< 0.001	2.20 (1.46–3.32)	< 0.001
Elevated CEA & normal CA19-9	1.76 (1.51–2.05)	< 0.001	1.70 (1.46–1.98)	< 0.001	1.70 (1.45–2.01)	< 0.001	1.90 (1.52–2.38)	< 0.001
Elevated CEA & elevated CA19-9	2.89 (2.40–3.48)	< 0.001	2.75 (2.28–3.32)	< 0.001	2.62 (2.14–3.21)	< 0.001	3.23 (2.46–4.24)	< 0.001

Note: *CA 19–9* carbohydrate antigen 19–9, *CEA* carcinoembryonic antigen, *OS* overall survival, *RFS* recurrence-free survival

Elevated CEA ≥ 5 ng/ml, normal CEA < 5 ng/ml; elevated CA 19–9 ≥ 37 U/ml, normal CA 19–9 < 37 U/ml

Model 1 was unadjusted. Model 2 was adjusted for sex (female vs. male), age. Model 3 was adjusted for sex (female vs. male), age, primary site (rectum vs. colon), surgical approach (open resection vs. laparoscopic resection), pathology stage (III → II), lymph node yield (≥ 12 vs. < 12), tumor differentiation (poor-undifferentiated vs. moderate vs. well), mucinous (colloid) type (yes vs. no), lymphovascular invasion / perineural invasion (yes vs. no), adjuvant chemotherapy (yes vs. no). Model 4 was adjusted for sex (female vs. male), age, primary site (rectum vs. colon), surgical approach (open resection vs. laparoscopic resection), pathology stage (III → II), lymph node yield (≥ 12 vs. < 12), tumor differentiation (poor-undifferentiated vs. moderate vs. well), mucinous (colloid) type (yes vs. no), lymphovascular invasion / perineural invasion (yes vs. no), adjuvant chemotherapy (yes vs. no), microsatellite instability (yes vs. no)

### Sensitivity analysis

The association between elevated preoperative CA19-9 with poorer RFS and OS in the overall population remained in the frailty model analysis (HR: 2.04, 95% CI: 1.81–2.31, *P* < 0.001; HR: 2.36, 95% CI: 2.03–2.74, *P* < 0.001) (Supplementary Table S9). Repeat analyses using the 74.0 U/mL CA19-9 cutoff produced similar results both before and after adjustment (Supplementary Tables S10).

### Subgroup analysis and cohort validation

Subgroup analysis of RFS and OS also found the elevated preoperative CA19-9 was associated with poor RFS and OS and absolute HRs varied in preoperative CEA strata (Supplementary Figure S4 and S5). There was no interaction between CA19-9 with other clinicopathologic factors known to be associated with prognosis in CRC patients. Separate analysis using data contained from the five cohorts yielded similar results (Supplementary Figure S4 and S5).

### Analysis of stage II CRC patients

The association between elevated preoperative CA19-9 with poorer RFS and OS in patients with stage II CRC (*n* = 2724) was maintained (unadjusted HR: 1.91, 95% CI: 1.54–2.36, *P* < 0.001; unadjusted HR: 1.98, 95% CI: 1.49–2.63, *P* < 0.001). In the analysis that included stage II CRC with normal preoperative CEA only, the 3-year RFS was 69.0% (59.7%-79.9%) vs. 85.5% (83.6%-87.3%) in patients with elevated vs. normal CA19-9 (unadjusted HR: 2.56, 95% CI: 1.72–3.83, *P* < 0.001). In the analysis that included stage II CRC with elevated preoperative CEA only, the 3-year RFS was 71.4% (65.5%-77.9%) vs. 80.5% (77.7%-83.5%) in patients with elevated vs. normal CA19-9 (unadjusted HR: 1.58, 95% CI: 1.19–2.11, *P* < 0.001) (Supplementary Figure S6a). The association remained after adjustment for risk factors that are known to affect survival in patients with stage II CRC. Analysis of OS produced similar trend, albeit not statistically significant (Supplementary Figure S6b). The adjuvant chemotherapy was not associated with favorable RFS in both stage II CRC subgroup with normal preoperative CA19-9 (unadjusted HR: 1.04, 95% CI: 0.85–1.26, *P* = 0.715) and elevated preoperative CA19-9 (HR: 1.41, 95% CI: 0.91–2.20, *P* = 0.126) (Supplementary Tables S11).

## Discussion

To our knowledge, this is the largest cohort study that examined the prognostic value of preoperative CA19-9 in CRC patients. The results from the current study confirmed that elevated serum preoperative CA19-9 is an independent risk for poor prognosis in CRC patients at stage II and III. When evaluating the results by subgroups and different cohorts, we found the similar results. Hence, our data supports that preoperative CA19-9 is an independent prognostic factor for CRC patients [10–19].

Serum tumor markers play an important role in prognosis prediction of CRC due to their the convenience of measurement. CEA is a recognized prognostic tumor marker in CRC, and current CRC guidelines recommend routine measurement of preoperative CEA [7, 20, 21]. However, as a commonly used serum tumor marker in CRC, the prognostic value of CA19-9 in CRC remains controversial. Most of previous studies have confirmed the independent prognostic role of preoperative CA19-9 in CRC, and suggested CA19-9 an additional marker to determine the prognosis of CRC patients without elevated preoperative CEA [11, 17, 27], which were concordant with our conclusion. Several studies have reported opposite results, concluding that CA19-9 could not provide more prognostic information than CEA [23, 35]. Currently, Chinese Society of Clinical Oncology include CEA and CA19-9 measurements in the Class II recommendation for the staging and prognostic stratification of colonoscopy-diagnosed CRC patients [7]. However, European Group on Tumour Markers [36] and American Society of Clinical Oncology [37] guidelines consider that the available evidence is insufficient to recommend CA19-9 for prognosis prediction in patients with CRC. For the controversy over the prognostic value of CA19-9, this study provides a multicenter, large-scale longitudinal cohort evidence.

We found also significant interaction between preoperative CA19-9 and CEA for their impact on the prognosis in the entire study population as well as in the five cohorts. The prognostic impact of CA19-9 varied in different preoperative CEA levels. The HR in patients with elevated versus normal CA19-9 for both RFS and OS is higher in patients with elevated CEA than in those with normal CEA. These findings suggest that the impact of preoperative CA19-9 on prognosis should be interpreted within the context of CEA in CRC patients.

We also showed that the combined effect of elevated preoperative CA19-9 and elevated preoperative CEA was higher than expected from the independent effects of both factors, as patients with both elevated CA19-9 level and elevated CEA level had approximately three times higher risk of recurrence compared to patients with neither of these conditions. This indicates CA19-9 and CEA may have a synergistic effect on CRC outcome.

CA19-9 is approved by the FDA as a biomarker in routine management in pancreatic cancer but not in CRC [38]. Preoperative CA19-9 has not been widely used prior to CRC surgery despite its availability [1–6], and current CRC guidelines do not support the routine use of CA19-9 for preoperative assessment [7–9]. This may be because whether preoperative CA19-9 is an independent prognostic factor for CRC patients remains controversial, and these are no multicenter studies with large sample sizes [10–26]. Fortunately, we in the present study showed that the preoperative CA19-9 is a prognostic biomarker in CRC, and our results have further confirmed in a large cohort the routine use of CA19-9 for preoperative assessment.

In the current study, preoperative CA19-9 was alone sufficient to classify stage II CRC patients into low- vs. high-risk groups. Unlike in previous study [19], multivariable analyses in the current study showed that preoperative CA19-9 was an independent predictor of RFS, even for CRC with MSI features. Such a discrepancy may be related to sample size differences. Also, stage II CRC patients with elevated preoperative CEA tended not to respond to adjuvant chemotherapy, possibly due to the variability of the adjuvant treatment. Prospectively designed cohort studies are needed to verify whether preoperative CA19-9 is helpful in predicting minimal residual disease after surgery.

This study is based on a cohort with large sample size and from multiple cancer research centers and hospitals. The results may represent the real-world situation. However, a limitation is the slight variations of different CA19-9 immunoassays across the five cancer centers and hospitals, and a lack of information for consistency among these assays. However, a sensitivity analysis using a higher cutoff value for elevated CA19-9 confirmed the association between elevated CA19-9 with poor prognosis, supporting the robustness of the finding. Other factors that are associated with serum CA19-9 and patient prognosis, such as tobacco use and Lewis antibody [39], were not fully controlled, as these were hard to truthfully ascertain from patients.

## Conclusions

In summary, our study has confirmed the prognostic value of serum preoperative CA19-9 in stage II-III CRC. Also, the prognostic impact of CA19-9 varied in different preoperative CEA levels. These findings encourage routine assessment of serum CA19-9 prior to CRC resection.

## Supplementary Information

**Additional file 1:** **FigureS1.** Association between preoperative CA19-9 status and overall survival. (a) overall population. (b) patients with normal preoperative CEA. (c) patientswith elevated preoperative CEA. Solid yellow lines are unadjustedhazard ratios, with dashed yellow lines showing 95% confidence intervalsderived from restricted cubic spline regressions. Reference lines for noassociation are indicated by the solid bold lines at a hazard ratio (HR) of 1.0. Dashed blue curves show the fraction of the population with different levels of preoperative CA19-9. Arrows indicate the concentration of preoperative CA19-9 with HR of 1.0. CA19-9, carbohydrate antigen 19-9; CEA, carcinoembryonic antigen; CI, confidence interval; E, number of events; HR, hazard ratio; N, number of patients. **FigureS2.** Kaplan‐Meier curves for overall survival according to the preoperative CA19-9 group. (a) overall population. (b) patients with normal preoperative CEA. (c) patientswith elevated preoperative CEA. CA19-9, carbohydrate antigen 19-9; CEA, carcinoembryonic antigen. **FigureS3.** Kaplan‐Meier curves according to the joint group of preoperative CEA and CA19-9 in colorectal cancer patients. (a) recurrence-free survival. (b) overall survival. CA19-9, carbohydrate antigen 19-9; CEA, carcinoembryonic antigen; OS, overall survival; RFS, recurrence-free survival. **FigureS4.** Forest plot for recurrence-free survival of preoperative CA 19-9 groups stratified by clinicopathological features based on the Cox models. *P* values for interaction were calculated using Cox regression model. HR and 95%CIs were given and visually represented by the squares and error bars. CA 19-9, carbohydrate antigen 19-9; CEA, carcinoembryonic antigen; CI, confidence interval; HR, hazard ratio. **FigureS5. **Forest plot for performance overallsurvival of preoperative CA19-9 groups stratified by clinicopathological features based on the Cox models. *P* values for interaction were calculated using Cox regression model. HR and 95%CIs were given and visually represented by the squares and error bars. CA19-9, carbohydrate antigen 19-9; CEA, carcinoembryonic antigen; CI, confidenceinterval; HR, hazard ratio. **FigureS6.** Kaplan‐Meier curves according to the joint group of preoperative CEA and CA19-9 in patients with stage II colorectal cancer. (a) recurrence-free survival.(b) overall survival. CA 19-9, carbohydrate antigen 19-9;CEA, carcinoembryonic antigen; OS, overall survival; RFS, recurrence-freesurvival.

**Additional file 2:** **Table S1**.Baseline characteristics by participant site. **Table S2**.Multivariate analyses of recurrence-free survival in total population (Cox model). **Table S3**.Multivariate analyses of overall survival in total population (Cox model). **Table S4**.Interaction between preoperative CEA and CA19-9 with risk of outcomes. **Table S5**.Multivariate analyses of recurrence-free survival in colorectal cancer subgroup with CEA < 5 ng/ml (Cox model). **Table S6**.Multivariate analyses of recurrence-free survival in colorectal cancer subgroup with CEA ≥ 5 ng/ml (Cox model). **Table S7**. Multivariate analyses of overall survival in colorectal cancer subgroup with CEA < 5 ng/ml (Cox model). **Table S8**.Multivariate analyses of overall survival in colorectal cancer subgroup with CEA ≥ 5 ng/ml (Cox model). **Table S9**.A frailty model analysis of preoperative CA19-9 (cutoff: 37 U/ml) on colorectal cancer outcomes in total population. **TableS10**.Cox proportional hazard regression analysis of preoperative CA19-9 (cutoff:74 U/ml) on colorectal cancer outcomes in total population. **Table S11**.Relationship between preoperative CA19-9 and benefit from adjuvant chemotherapyin patients with stage II colorectal cancer.

## Data Availability

The data underlying this article cannot be shared publicly due to individuals’ privacy that participated in the study. The data will be shared on a reasonable request to the corresponding author.

## Abbreviations

CRC: Colorectal cancer
CEA: Carcinoembryonic antigen
CA19-9: Carbohydrate antigen 19–9
MSI: Microsatellite instability
RCS: Restricted cubic spline
IQR: Interquartile range
OS: Overall survival
RFS: Recurrence-free survival
HR: Hazard ratio
CI: Confidence Interval
REMARK: Reporting Recommendations for Tumor Marker Prognostic Studies
STROBE: Strengthening the Reporting of Observational Studies in Epidemiology
