# Supplementary material for: Preoperative serum CA19-9 should be routinely measured in the colorectal patients with preoperative normal serum CEA: a multicenter retrospective cohort study
Source: BMC Cancer. 2022 Sep 8;22:962. doi: 10.1186/s12885-022-10051-2 (PMC9454113; doi:10.1186/s12885-022-10051-2)
Supplement: Supplementary file 2 — Additional file 2: Table S1.Baseline characteristics by participant site. Table S2.Multivariate analyses of recurrence-free survival in total population (Cox model). Table S3.Multivariate analyses of overall survival in total population (Cox model). Table S4.Interaction between preoperative CEA and CA19-9 with risk of outcomes. Table S5.Multivariate analyses of recurrence-free survival in colorectal cancer subgroup with CEA < 5 ng/ml (Cox model). Table S6.Multivariate analyses of recurrence-free survival in colorectal cancer subgroup with CEA ≥ 5 ng/ml (Cox model). Table S7. Multivariate analyses of overall survival in colorectal cancer subgroup with CEA < 5 ng/ml (Cox model). Table S8.Multivariate analyses of overall survival in colorectal cancer subgroup with CEA ≥ 5 ng/ml (Cox model). Table S9.A frailty model analysis of preoperative CA19-9 (cutoff: 37 U/ml) on colorectal cancer outcomes in total population. TableS10.Cox proportional hazard regression analysis of preoperative CA19-9 (cutoff:74 U/ml) on colorectal cancer outcomes in total population. Table S11.Relationship between preoperative CA19-9 and benefit from adjuvant chemotherapyin patients with stage II colorectal cancer. [file 12885_2022_10051_MOESM2_ESM.docx]

**Table S1**. Baseline characteristics by participant site

| **Variable** | **YNCH (n = 2170)** | **KMU1 (n = 1111)** | **PUCH (n = 604)** | **SYSU6 (n = 683)** | **CQU1 (n = 480)** | **Total (n = 5048)** | ***P* value** |
| --- | --- | --- | --- | --- | --- | --- | --- |
| Male, n (%) | 1301 (60.0) | 662 (59.6) | 375 (62.1) | 401 (58.7) | 290 (60.4) | 3029 (60.0) | 0.794 |
| Age^a^ | 60.0 [51.0, 68.0] | 61.0 [51.0, 69.0] | 63.0 [54.0, 70.0] | 58.0 [49.0, 65.0] | 64.0 [53.8, 71.0] | 61.0 [51.0, 68.0] | < 0.001 |
| Preoperative CA19-9, U/ml ^a^ | 13.3 [7.8, 24.8] | 11.5 [8.3, 18.3] | 11.9 [7.6, 21.3] | 10.5 [4.8, 26.5] | 13.1 [7.2, 27.6] | 11.9 [7.3, 23.4] | 0.001 |
| Preoperative CA19-9 group, n (%) |  |  |  |  |  |  | < 0.001 |
| ≥ 37 U/ml | 327 (15.1) | 125 (11.3) | 63 (10.4) | 138 (20.2) | 85 (17.7) | 738 (14.6) |  |
| < 37 U/ml | 1843 (84.9) | 986 (88.7) | 541 (89.6) | 545 (79.8) | 395 (82.3) | 4310 (85.4) |  |
| Preoperative CEA, ng/ml ^a^ | 4.3 [2.3, 10.8] | 3.5 [2.1, 7.9] | 3.2 [1.8, 7.2] | 3.5 [2.0, 10.0] | 4.3 [2.0, 9.6] | 3.8 [2.1, 9.4] | < 0.001 |
| Preoperative CEA group, n (%) |  |  |  |  |  |  | < 0.001 |
| ≥ 5 ng/ml | 976 (45.0) | 385 (34.7) | 207 (34.3) | 268 (39.2) | 207 (43.1) | 2043 (40.5) |  |
| < 5 ng/ml | 1194 (55.0) | 726 (65.3) | 397 (65.7) | 415 (60.8) | 273 (56.9) | 3005 (59.5) |  |
| Primary site, n (%) |  |  |  |  |  |  | 0.001 |
| Colon | 1153 (53.1) | 594 (53.5) | 305 (50.5) | 391 (57.2) | 216 (45.0) | 2659 (52.7) |  |
| Rectum | 1017 (46.9) | 517 (46.5) | 299 (49.5) | 292 (42.8) | 264 (55.0) | 2389 (47.3) |  |
| Surgical approach, n (%) |  |  |  |  |  |  | < 0.001 |
| Laparoscopic resection | 791 (36.5) | 914 (82.3) | 265 (43.9) | 590 (86.4) | 450 (93.8) | 3010 (59.6) |  |
| Open resection | 1379 (63.5) | 197 (17.7) | 336 (55.6) | 93 (13.6) | 30 (6.2) | 2035 (40.3) |  |
| Unknown | 0 (0.0) | 0 (0.0) | 3 (0.5) | 0 (0.0) | 0 (0.0) | 3 (0.1) |  |
| AJCC 8th ed. Stage, n (%) |  |  |  |  |  |  | < 0.001 |
| II | 1108 (51.1) | 592 (53.3) | 499 (82.6) | 287 (42.0) | 238 (49.6) | 2724 (54.0) |  |
| III | 1062 (48.9) | 519 (46.7) | 105 (17.4) | 396 (58.0) | 242 (50.4) | 2324 (46.0) |  |
| Lymph node yield, n (%) |  |  |  |  |  |  | < 0.001 |
| ≥ 12 | 2201 (78.6) | 962 (69.4) | 411 (45.4) | 701 (88.6) | 441 (77.6) | 4716 (73.1) |  |
| < 12 | 601 (21.4) | 424 (30.6) | 495 (54.6) | 89 (11.3) | 124 (21.8) | 1733 (26.9) |  |
| Unknown | 0 (0.0) | 0 (0.0) | 0 (0.0) | 1 (0.1) | 3 (0.5) | 4 (0.1) |  |
| Tumor differentiation, n (%) |  |  |  |  |  |  | < 0.001 |
| Well-moderate | 1353 (62.4) | 952 (85.7) | 294 (48.7) | 518 (75.8) | 416 (86.7) | 3533 (70.0) |  |
| Poor-undifferentiated | 709 (32.7) | 159 (14.3) | 11 (1.8) | 106 (15.5) | 55 (11.5) | 1040 (20.6) |  |
| Unknown | 108 (5.0) | 0 (0.0) | 299 (49.5) | 59 (8.6) | 9 (1.9) | 475 (9.4) |  |
| Mucinous (colloid) type, n (%) |  |  |  |  |  |  | < 0.001 |
| Yes | 135 (6.2) | 32 (2.9) | 66 (10.9) | 49 (7.2) | 113 (23.5) | 395 (7.8) |  |
| No | 2035 (93.8) | 1079 (97.1) | 535 (88.6) | 634 (92.8) | 365 (76.0) | 4648 (92.1) |  |
| Unknown | 0 (0.0) | 0 (0.0) | 3 (0.5) | 0 (0.0) | 2 (0.4) | 5 (0.1) |  |
| Lymphovascular / Perineural invasion, n (%) |  |  |  |  |  |  | < 0.001 |
| Yes | 219 (10.1) | 582 (52.4) | 165 (27.3) | 166 (24.3) | 36 (7.5) | 1168 (23.1) |  |
| No | 1951 (89.9) | 529 (47.6) | 439 (72.7) | 514 (75.3) | 326 (67.9) | 3759 (74.5) |  |
| Unknown | 0 (0.0) | 0 (0.0) | 0 (0.0) | 3 (0.4) | 118 (24.6) | 121 (2.4) |  |
| Adjuvant chemotherapy, n (%) |  |  |  |  |  |  | < 0.001 |
| Yes | 1529 (70.5) | 909 (81.8) | 334 (55.3) | 567 (83.0) | 237 (49.4) | 3576 (70.8) |  |
| No | 641 (29.5) | 202 (18.2) | 270 (44.7) | 115 (16.8) | 243 (50.6) | 1471 (29.1) |  |
| Unknown | 0 (0.0) | 0 (0.0) | 0 (0.0) | 1 (0.1) | 0 (0.0) | 1 (0.0) |  |
| MSI, n (%) |  |  |  |  |  |  | < 0.001 |
| Yes | 33 (1.5) | 304 (27.4) | 84 (13.9) | 58 (8.5) | 407 (84.8) | 886 (17.6) |  |
| No | 397 (18.3) | 807 (72.6) | 459 (76.0) | 620 (90.8) | 71 (14.8) | 2354 (46.6) |  |
| Unknown | 1740 (80.2) | 0 (0.0) | 61 (10.1) | 5 (0.7) | 2 (0.4) | 1808 (35.8) |  |

Note: ^a^, Data is median [IQR].

CA 19-9, carbohydrate antigen 19-9; CEA, carcinoembryonic antigen; MSI, microsatellite instability.

CQU1, the First Affiliated Hospital of Chongqing Medical University; KMU1, the First Affiliated Hospital of Kunming Medical University; PUCH, Peking University Cancer Hospital & Institute; SYSU6, the Sixth Affiliated Hospital of Sun Yat-sen University; YNCH, Yunnan Cancer Hospital.

**Table S2**. Multivariate analyses of recurrence-free survival in total population (Cox model)

|  | Model 1 | Model 2 | Model 3 | Model 4 |
| --- | --- | --- | --- | --- |
| CA19-9 groups |  |  |  |  |
| CA19-9 < 37 ng/ml | Reference | Reference | Reference | Reference |
| CA19-9 ≥ 37 ng/ml | 2.08 (1.84-2.36) | 2.08 (1.84-2.36) | 1.90 (1.67-2.16) | 2.08 (1.75-2.47) |
| Covariates |  |  |  |  |
| Sex |  |  |  |  |
| Male |  | Reference | Reference | Reference |
| Female |  | 0.94 (0.84-1.04) | 0.92 (0.82-1.02) | 0.82 (0.71-0.95) |
| Age |  | 1.01 (1.00-1.01) | 1.01 (1.00-1.01) | 1.02 (1.01-1.02) |
| Primary site |  |  |  |  |
| Colon |  |  | Reference | Reference |
| Rectum |  |  | 1.27 (1.13-1.42) | 1.31 (1.13-1.53) |
| Surgical approach |  |  |  |  |
| Laparoscopic resection |  |  | Reference | Reference |
| Open resection |  |  | 1.22 (1.09-1.37) | 1.38 (1.16-1.64) |
| AJCC 8th ed. Stage |  |  |  |  |
| II |  |  | Reference | Reference |
| III |  |  | 2.00 (1.78-2.25) | 1.81 (1.55-2.12) |
| Lymph node yield |  |  |  |  |
| ≥12 |  |  | Reference | Reference |
| <12 |  |  | 1.07 (0.94-1.22) | 1.06 (0.90-1.25) |
| Tumor differentiation |  |  |  |  |
| Well-moderate |  |  | Reference | Reference |
| Poor-undifferentiated |  |  | 1.30 (1.15-1.47) | 1.54 (1.29-1.86) |
| Mucinous (colloid) type |  |  |  |  |
| No |  |  | Reference | Reference |
| Yes |  |  | 1.12 (0.89-1.41) | 1.02 (0.78-1.35) |
| Lymphovascular / Perineural invasion |  |  |  |  |
| No |  |  | Reference | Reference |
| Yes |  |  | 1.79 (1.59-2.01) | 1.73 (1.50-2.01) |
| Adjuvant chemotherapy |  |  |  |  |
| No |  |  | Reference | Reference |
| Yes |  |  | 0.93 (0.81-1.06) | 1.07 (0.89-1.29) |
| Microsatellite instability |  |  |  |  |
| No |  |  |  | Reference |
| Yes |  |  |  | 1.30 (1.11-1.52) |

Note: CA 19-9, carbohydrate antigen 19-9; CEA, carcinoembryonic antigen.

**Table S3**. Multivariate analyses of overall survival in total population (Cox model)

|  | Model 1 | Model 2 | Model 3 | Model 4 |
| --- | --- | --- | --- | --- |
| CA199 groups |  |  |  |  |
| CA199 < 37 ng/ml | Reference | Reference | Reference | Reference |
| CA199 ≥ 37 ng/ml | 2.28 (1.96-2.65) | 2.26 (1.95-2.63) | 2.05 (1.74-2.42) | 2.25 (1.80-2.81) |
| Covariates |  |  |  |  |
| Sex |  |  |  |  |
| Male |  | Reference | Reference | Reference |
| Female |  | 0.95 (0.83-1.09) | 0.92 (0.80-1.06) | 0.87 (0.71-1.06) |
| Age |  | 1.02 (1.01-1.02) | 1.02 (1.01-1.02) | 1.03 (1.02-1.04) |
| Primary site |  |  |  |  |
| Colon |  |  | Reference | Reference |
| Rectum |  |  | 1.29 (1.11-1.49) | 1.37 (1.12-1.68) |
| Surgical approach |  |  |  |  |
| Laparoscopic resection |  |  | Reference | Reference |
| Open resection |  |  | 1.33 (1.15-1.55) | 1.45 (1.15-1.82) |
| AJCC 8th ed. Stage |  |  |  |  |
| II |  |  | Reference | Reference |
| III |  |  | 2.43 (2.08-2.84) | 2.10 (1.69-2.61) |
| Lymph node yield |  |  |  |  |
| ≥12 |  |  | Reference | Reference |
| <12 |  |  | 1.12 (0.95-1.32) | 1.21 (0.95-1.5) |
| Tumor differentiation |  |  |  |  |
| Well-moderate |  |  | Reference | Reference |
| Poor-undifferentiated |  |  | 1.39 (1.19-1.63) | 1.77 (1.39-2.25) |
| Mucinous (colloid) type |  |  |  |  |
| No |  |  | Reference | Reference |
| Yes |  |  | 0.94 (0.69-1.29) | 0.73 (0.48-1.10) |
| Lymphovascular / Perineural invasion |  |  |  |  |
| No |  |  | Reference | Reference |
| Yes |  |  | 2.16 (1.85-2.51) | 2.12 (1.74-2.58) |
| Adjuvant chemotherapy |  |  |  |  |
| No |  |  | Reference | Reference |
| Yes |  |  | 0.69 (0.59-0.82) | 0.85 (0.66-1.08) |
| Microsatellite instability |  |  |  |  |
| No |  |  |  | Reference |
| Yes |  |  |  | 1.26 (1.01-1.56) |

Note: CA 19-9, carbohydrate antigen 19-9; CEA, carcinoembryonic antigen.

**Table S4**. Interaction between preoperative CEA and CA19-9 with risk of outcomes

| Outcome | Preoperative CEA^a^ | |  | Preoperative CA19-9^b^ | |  | Interaction between Preoperative CEA^a^ and CA19-9^b^ | |
| --- | --- | --- | --- | --- | --- | --- | --- | --- |
|  | Hazard Ratio (95% CI) | *P* Value |  | Hazard Ratio (95% CI) | *P* Value |  | Hazard Ratio (95% CI) | *P* Value |
| RFS |  |  |  |  |  |  |  |  |
| Model1 | 1.54 (1.37-1.73) | < 0.001 |  | 2.32 (1.87-2.88) | < 0.001 |  | 0.67 (0.52-0.88) | 0.003 |
| Model2 | 1.52 (1.35-1.71) | < 0.001 |  | 2.38 (1.92-2.96) | < 0.001 |  | 0.66 (0.50-0.86) | 0.002 |
| Model3 | 1.50 (1.32-1.70) | < 0.001 |  | 2.08 (1.65-2.62) | < 0.001 |  | 0.74 (0.56-0.98) | 0.036 |
| Model4 | 1.65 (1.40-1.95) | < 0.001 |  | 2.00 (1.46-2.72) | < 0.001 |  | 0.84 (0.58-1.22) | 0.351 |
| OS |  |  |  |  |  |  |  |  |
| Model1 | 1.76 (1.51-2.05) | < 0.001 |  | 2.85 (2.18-3.72) | < 0.001 |  | 0.58 (0.42-0.80) | 0.001 |
| Model2 | 1.70 (1.46-1.98) | < 0.001 |  | 3.02 (2.31-3.94) | < 0.001 |  | 0.54 (0.39-0.74) | < 0.001 |
| Model3 | 1.70 (1.45-2.01) | < 0.001 |  | 2.52 (1.88-3.37) | < 0.001 |  | 0.61 (0.43-0.87) | 0.006 |
| Model4 | 1.90 (1.52-2.38) | < 0.001 |  | 2.20 (1.46-3.32) | < 0.001 |  | 0.77 (0.47-1.27) | 0.306 |

Note: CA 19-9, carbohydrate antigen 19-9; CEA, carcinoembryonic antigen; CI, confidence interval; OS, overall survival; RFS, recurrence-free survival.

Model 1 was unadjusted. Model 2 was adjusted for sex (female vs. male), age. Model 3 was adjusted for sex (female vs. male), age, primary site (rectum vs. colon), surgical approach (open resection vs. laparoscopic resection), pathology stage (III→II), lymph node yield (≥12 vs. <12), tumor differentiation (poor-undifferentiated vs. moderate vs. well), mucinous (colloid) type (yes vs. no), lymphovascular invasion / perineural invasion (yes vs. no), adjuvant chemotherapy (yes vs. no). Model 4 was adjusted for sex (female vs. male), age, primary site (rectum vs. colon), surgical approach (open resection vs. laparoscopic resection), pathology stage (III→II), lymph node yield (≥12 vs. <12), tumor differentiation (poor-undifferentiated vs. moderate vs. well), mucinous (colloid) type (yes vs. no), lymphovascular invasion / perineural invasion (yes vs. no), adjuvant chemotherapy (yes vs. no) , microsatellite instability (yes vs. no).

^a^, elevated CEA (≥ 5 ng/ml) vs. normal CEA (< 5 ng/ml); ^b^, elevated CA 19-9 (≥ 37 ng/ml) vs. normal CA 19-9 (< 37 ng/ml).

**Table S5**. Multivariate analyses of recurrence-free survival in colorectal cancer subgroup with CEA < 5 ng/ml (Cox model)

|  | Model 1 | Model 2 | Model 3 | Model 4 |
| --- | --- | --- | --- | --- |
| CA199 groups |  |  |  |  |
| CA199 < 37 ng/ml | Reference | Reference | Reference | Reference |
| CA199 ≥ 37 ng/ml | 2.34 (1.89-2.90) | 2.41 (1.94-2.99) | 2.01 (1.66-2.66) | 2.01 (1.47-2.74) |
| Covariates |  |  |  |  |
| Sex |  |  |  |  |
| Male |  | Reference | Reference | Reference |
| Female |  | 0.93 (0.80-1.08) | 0.92 (0.78-1.08) | 0.79 (0.63-0.97) |
| Age |  | 1.01 (1.00-1.01) | 1.01 (1.00-1.02) | 1.02 (1.01-1.03) |
| Primary site |  |  |  |  |
| Colon |  |  | Reference | Reference |
| Rectum |  |  | 1.34 (1.14-1.57) | 1.36 (1.10-1.69) |
| Surgical approach |  |  |  |  |
| Laparoscopic resection |  |  | Reference | Reference |
| Open resection |  |  | 1.21 (1.03-1.43) | 1.16 (0.89-1.50) |
| AJCC 8th ed. Stage |  |  |  |  |
| II |  |  | Reference | Reference |
| III |  |  | 1.88 (1.59-2.23) | 1.73 (1.38-2.17) |
| Lymph node yield |  |  |  |  |
| ≥12 |  |  | Reference | Reference |
| <12 |  |  | 1.00 (0.83-1.20) | 0.96 (0.76-1.21) |
| Tumor differentiation |  |  |  |  |
| Well-moderate |  |  | Reference | Reference |
| Poor-undifferentiated |  |  | 1.38 (1.16-1.64) | 1.58 (1.22-2.03) |
| Mucinous (colloid) type |  |  |  |  |
| No |  |  | Reference | Reference |
| Yes |  |  | 1.24 (0.91-1.70) | 1.27 (0.88-1.84) |
| Lymphovascular / Perineural invasion |  |  |  |  |
| No |  |  | Reference | Reference |
| Yes |  |  | 1.62 (1.37-1.93) | 1.65 (1.34-2.04) |
| Adjuvant chemotherapy |  |  |  |  |
| No |  |  | Reference | Reference |
| Yes |  |  | 0.94 (0.77-1.13) | 1.08 (0.83-1.40) |
| Microsatellite instability |  |  |  |  |
| No |  |  |  | Reference |
| Yes |  |  |  | 1.35 (1.08-1.69) |

Note: CA 19-9, carbohydrate antigen 19-9; CEA, carcinoembryonic antigen.

**Table S6**. Multivariate analyses of recurrence-free survival in colorectal cancer subgroup with CEA ≥ 5 ng/ml (Cox model)

|  | Model 1 | Model 2 | Model 3 | Model 4 |
| --- | --- | --- | --- | --- |
| CA199 groups |  |  |  |  |
| CA199 < 37 ng/ml | Reference | Reference | Reference | Reference |
| CA199 ≥ 37 ng/ml | 1.56 (1.34-1.82) | 1.56 (1.34-1.82) | 1.54 (1.30-1.81) | 1.68 (1.35-2.08) |
| Covariates |  |  |  |  |
| Sex |  |  |  |  |
| Male |  | Reference | Reference | Reference |
| Female |  | 0.94 (0.81-1.08) | 0.92 (0.78-1.07) | 0.88 (0.71-1.08) |
| Age |  | 1.00 (0.998-1.01) | 1.00 (0.997-1.01) | 1.01 (1.00-1.02) |
| Primary site |  |  |  |  |
| Colon |  |  | Reference | Reference |
| Rectum |  |  | 1.19 (1.02-1.39) | 1.24 (1.00-1.54) |
| Surgical approach |  |  |  |  |
| Laparoscopic resection |  |  | Reference | Reference |
| Open resection |  |  | 1.16 (0.992-1.37) | 1.54 (1.21-1.94) |
| AJCC 8th ed. Stage |  |  |  |  |
| II |  |  | Reference | Reference |
| III |  |  | 2.12 (1.79-2.49) | 1.98 (1.59-2.47) |
| Lymph node yield |  |  |  |  |
| ≥12 |  |  | Reference | Reference |
| <12 |  |  | 1.21 (1.01-1.45) | 1.25 (0.99-1.57) |
| Tumor differentiation |  |  |  |  |
| Well-moderate |  |  | Reference | Reference |
| Poor-undifferentiated |  |  | 1.24 (1.04-1.48) | 1.63 (1.24-2.14) |
| Mucinous (colloid) type |  |  |  |  |
| No |  |  | Reference | Reference |
| Yes |  |  | 1.00 (0.71-1.41) | 0.76 (0.50-1.16) |
| Lymphovascular / Perineural invasion |  |  |  |  |
| No |  |  | Reference | Reference |
| Yes |  |  | 1.99 (1.69-2.35) | 1.89 (1.54-2.33) |
| Adjuvant chemotherapy |  |  |  |  |
| No |  |  | Reference | Reference |
| Yes |  |  | 0.91 (0.75-1.09) | 1.00 (0.76-1.30) |
| Microsatellite instability |  |  |  |  |
| No |  |  |  | Reference |
| Yes |  |  |  | 1.30 (1.03-1.64) |

Note: CA 19-9, carbohydrate antigen 19-9; CEA, carcinoembryonic antigen.

**Table S7**. Multivariate analyses of overall survival in colorectal cancer subgroup with CEA < 5 ng/ml (Cox model)

|  | Model 1 | Model 2 | Model 3 | Model 4 |
| --- | --- | --- | --- | --- |
| CA199 groups |  |  |  |  |
| CA199 < 37 ng/ml | Reference | Reference | Reference | Reference |
| CA199 ≥ 37 ng/ml | 2.85 (2.18-3.72) | 3.02 (2.31-3.95) | 2.54 (1.89-3.42) | 2.20 (1.44-3.35) |
| Covariates |  |  |  |  |
| Sex |  |  |  |  |
| Male |  | Reference | Reference | Reference |
| Female |  | 1.00 (0.82-1.22) | 0.97 (0.78-1.20) | 0.75 (0.56-1.02) |
| Age |  | 1.02 (1.01-1.03) | 1.02 (1.01-1.03) | 1.03 (1.02-1.05) |
| Primary site |  |  |  |  |
| Colon |  |  | Reference | Reference |
| Rectum |  |  | 1.34 (1.08-1.67) | 1.28 (0.95-1.73) |
| Surgical approach |  |  |  |  |
| Laparoscopic resection |  |  | Reference | Reference |
| Open resection |  |  | 1.35 (1.08-1.68) | 1.25 (0.88-1.78) |
| AJCC 8th ed. Stage |  |  |  |  |
| II |  |  | Reference | Reference |
| III |  |  | 2.32 (1.84-2.92) | 2.03 (1.47-2.80) |
| Lymph node yield |  |  |  |  |
| ≥12 |  |  | Reference | Reference |
| <12 |  |  | 1.11 (0.87-1.41) | 1.15 (0.84-1.58) |
| Tumor differentiation |  |  |  |  |
| Well-moderate |  |  | Reference | Reference |
| Poor-undifferentiated |  |  | 1.60 (1.27-2.00) | 2.05 (1.46-2.88) |
| Mucinous (colloid) type |  |  |  |  |
| No |  |  | Reference | Reference |
| Yes |  |  | 1.08 (0.70-1.66) | 0.95 (0.55-1.64) |
| Lymphovascular / Perineural invasion |  |  |  |  |
| No |  |  | Reference | Reference |
| Yes |  |  | 1.79 (1.42-2.26) | 1.73 (1.29-2.31) |
| Adjuvant chemotherapy |  |  |  |  |
| No |  |  | Reference | Reference |
| Yes |  |  | 0.67 (0.52-0.85) | 0.81 (0.57-1.15) |
| Microsatellite instability |  |  |  |  |
| No |  |  |  | Reference |
| Yes |  |  |  | 1.24 (0.90-1.70) |

Note: CA 19-9, carbohydrate antigen 19-9; CEA, carcinoembryonic antigen.

**Table S8**. Multivariate analyses of overall survival in colorectal cancer subgroup with CEA ≥ 5 ng/ml (Cox model)

|  | Model 1 | Model 2 | Model 3 | Model 4 |
| --- | --- | --- | --- | --- |
| CA199 groups |  |  |  |  |
| CA199 < 37 ng/ml | Reference | Reference | Reference | Reference |
| CA199 ≥ 37 ng/ml | 1.64 (1.36-1.98) | 1.63 (1.35-1.97) | 1.55 (1.27-1.90) | 1.72 (1.30-2.28) |
| Covariates |  |  |  |  |
| Sex |  |  |  |  |
| Male |  | Reference | Reference | Reference |
| Female |  | 0.90 (0.75-1.08) | 0.90 (0.74-1.09) | 1.00 (0.77-1.31) |
| Age |  | 1.01 (1.01-1.02) | 1.01 (1.01-1.02) | 1.02 (1.01-1.03) |
| Primary site |  |  |  |  |
| Colon |  |  | Reference | Reference |
| Rectum |  |  | 1.22 (1.00-1.48) | 1.44 (1.08-1.91) |
| Surgical approach |  |  |  |  |
| Laparoscopic resection |  |  | Reference | Reference |
| Open resection |  |  | 1.21 (0.991-1.48) | 1.56 (1.15-2.13) |
| AJCC 8th ed. Stage |  |  |  |  |
| II |  |  | Reference | Reference |
| III |  |  | 2.51 (2.03-3.10) | 2.34 (1.74-3.15) |
| Lymph node yield |  |  |  |  |
| ≥12 |  |  | Reference | Reference |
| <12 |  |  | 1.22 (0.97-1.53) | 1.35 (1.01-1.81) |
| Tumor differentiation |  |  |  |  |
| Well-moderate |  |  | Reference | Reference |
| Poor-undifferentiated |  |  | 1.25 (1.01-1.55) | 1.60 (1.13-2.27) |
| Mucinous (colloid) type |  |  |  |  |
| No |  |  | Reference | Reference |
| Yes |  |  | 0.81 (0.51-1.30) | 0.57 (0.30-1.06) |
| Lymphovascular / Perineural invasion |  |  |  |  |
| No |  |  | Reference | Reference |
| Yes |  |  | 2.54 (2.08-3.10) | 2.63 (2.01-3.43) |
| Adjuvant chemotherapy |  |  |  |  |
| No |  |  | Reference | Reference |
| Yes |  |  | 0.70 (0.56-0.87) | 0.83 (0.59-1.16) |
| Microsatellite instability |  |  |  |  |
| No |  |  |  | Reference |
| Yes |  |  |  | 1.27 (0.94-1.72) |

Note: CA 19-9, carbohydrate antigen 19-9; CEA, carcinoembryonic antigen.

**Table S9**. A frailty model analysis of preoperative CA19-9 (cutoff: 37ng/ml) on colorectal cancer outcomes in total population

| Outcome | total | |  | CEA < 5 ng/ml | |  | CEA > 5 ng/ml | |
| --- | --- | --- | --- | --- | --- | --- | --- | --- |
|  | Hazard Ratio (95% CI) | *P* Value |  | Hazard Ratio (95% CI) | *P* Value |  | Hazard Ratio (95% CI) | *P* Value |
| RFS |  |  |  |  |  |  |  |  |
| Model1 | 2.04 (1.81-2.31) | < 0.001 |  | 2.43 (1.95-3.02) | < 0.001 |  | 1.57 (1.35-1.83) | < 0.001 |
| Model2 | 2.03 (1.80-2.30) | < 0.001 |  | 2.48 (1.99-3.08) | < 0.001 |  | 1.57 (1.34-1.83) | < 0.001 |
| Model3 | 1.92 (1.68-2.18) | < 0.001 |  | 2.17 (1.71-2.74) | < 0.001 |  | 1.54 (1.30-1.81) | < 0.001 |
| Model4 | 2.09 (1.76-2.48) | < 0.001 |  | 2.05 (1.49-2.81) | < 0.001 |  | 1.68 (1.35-2.08) | < 0.001 |
| OS |  |  |  |  |  |  |  |  |
| Model1 | 2.36 (2.03-2.74) | < 0.001 |  | 2.99 (2.29-3.92) | < 0.001 |  | 1.67 (1.38-2.02) | < 0.001 |
| Model2 | 2.33 (2.00-2.71) | < 0.001 |  | 3.12 (2.39-4.09) | < 0.001 |  | 1.66 (1.37-2.01) | < 0.001 |
| Model3 | 2.10 (1.78-2.48) | < 0.001 |  | 2.66 (1.97-3.59) | < 0.001 |  | 1.57 (1.28-1.92) | < 0.001 |
| Model4 | 2.31 (1.84-2.90) | < 0.001 |  | 2.30 (1.50-3.52) | < 0.001 |  | 1.74 (1.32-2.30) | < 0.001 |

Note: CA 19-9, carbohydrate antigen 19-9; CEA, carcinoembryonic antigen; CI, confidence interval; OS, overall survival; RFS, recurrence-free survival.

Model 1 was unadjusted. Model 2 was adjusted for sex (female vs. male), age. Model 3 was adjusted for sex (female vs. male), age, primary site (rectum vs. colon), surgical approach (open resection vs. laparoscopic resection), pathology stage (III→II), lymph node yield (≥12 vs. <12), tumor differentiation (poor-undifferentiated vs. moderate vs. well), mucinous (colloid) type (yes vs. no), lymphovascular invasion / perineural invasion (yes vs. no), adjuvant chemotherapy (yes vs. no). Model 4 was adjusted for sex (female vs. male), age, primary site (rectum vs. colon), surgical approach (open resection vs. laparoscopic resection), pathology stage (III→II), lymph node yield (≥12 vs. <12), tumor differentiation (poor-undifferentiated vs. moderate vs. well), mucinous (colloid) type (yes vs. no), lymphovascular invasion / perineural invasion (yes vs. no), adjuvant chemotherapy (yes vs. no) , microsatellite instability (yes vs. no).

**Table S10**. Cox proportional hazard regression analysis of preoperative CA19-9 (cutoff: 74ng/ml) on colorectal cancer outcomes in total population

| Outcome | total | |  | CEA < 5 ng/ml | |  | CEA ≥ 5 ng/ml | |
| --- | --- | --- | --- | --- | --- | --- | --- | --- |
|  | Hazard Ratio (95% CI) | *P* Value |  | Hazard Ratio (95% CI) | *P* Value |  | Hazard Ratio (95% CI) | *P* Value |
| RFS |  |  |  |  |  |  |  |  |
| Model1 | 2.26 (1.93-2.65) | < 0.001 |  | 2.82 (2.05-3.88) | < 0.001 |  | 1.73 (1.44-2.08) | < 0.001 |
| Model2 | 2.29 (1.96-2.68) | < 0.001 |  | 2.95 (2.14-4.07) | < 0.001 |  | 1.75 (1.45-2.11) | < 0.001 |
| Model3 | 2.10 (1.77-2.48) | < 0.001 |  | 2.60 (1.85-3.67) | < 0.001 |  | 1.65 (1.35-2.01) | < 0.001 |
| Model4 | 2.50 (2.01-3.12) | < 0.001 |  | 2.67 (1.69-4.22) | < 0.001 |  | 1.94 (1.49-2.51) | < 0.001 |
| OS |  |  |  |  |  |  |  |  |
| Model1 | 2.65 (2.19-3.20) | < 0.001 |  | 4.01 (2.78-5.78) | < 0.001 |  | 1.83 (1.46-2.30) | < 0.001 |
| Model2 | 2.72 (2.25-3.30) | < 0.001 |  | 4.52 (3.12-6.55) | < 0.001 |  | 1.89 (1.50-2.37) | < 0.001 |
| Model3 | 2.39 (1.94-2.94) | < 0.001 |  | 3.68 (2.46-5.52) | < 0.001 |  | 1.72 (1.34-2.20) | < 0.001 |
| Model4 | 3.02 (2.29-3.98) | < 0.001 |  | 3.42 (1.92-6.08) | < 0.001 |  | 2.14 (1.53-2.97) | < 0.001 |

Note: CA 19-9, carbohydrate antigen 19-9; CEA, carcinoembryonic antigen; CI, confidence interval; OS, overall survival; RFS, recurrence-free survival.

Model 1 was unadjusted. Model 2 was adjusted for sex (female vs. male), age. Model 3 was adjusted for sex (female vs. male), age, primary site (rectum vs. colon), surgical approach (open resection vs. laparoscopic resection), pathology stage (III→II), lymph node yield (≥12 vs. <12), tumor differentiation (poor-undifferentiated vs. moderate vs. well), mucinous (colloid) type (yes vs. no), lymphovascular invasion / perineural invasion (yes vs. no), adjuvant chemotherapy (yes vs. no). Model 4 was adjusted for sex (female vs. male), age, primary site (rectum vs. colon), surgical approach (open resection vs. laparoscopic resection), pathology stage (III→II), lymph node yield (≥12 vs. <12), tumor differentiation (poor-undifferentiated vs. moderate vs. well), mucinous (colloid) type (yes vs. no), lymphovascular invasion / perineural invasion (yes vs. no), adjuvant chemotherapy (yes vs. no), microsatellite instability (yes vs. no).

**Table S11**. Relationship between preoperative CA19-9 and benefit from adjuvant chemotherapy in patients with stage II colorectal cancer

|  | Model 1 | |  | Model 2 | |  | Model 3 | |  | Model 4 | |
| --- | --- | --- | --- | --- | --- | --- | --- | --- | --- | --- | --- |
|  | HR (95% CI) | P value |  | HR (95% CI) | P value |  | HR (95% CI) | P value |  | HR (95% CI) | P value |
| OS |  |  |  |  |  |  |  |  |  |  |  |
| Total | 0.76 (0.60-0.96) | 0.020 |  | 0.86 (0.67-1.10) | 0.226 |  | 0.84 (0.64-1.10) | 0.207 |  | 1.09 (0.76-1.56) | 0.642 |
| Normal CA19-9 | 0.69 (0.53-0.89) | 0.005 |  | 0.79 (0.60-1.03) | 0.087 |  | 0.76 (0.58-1.00) | 0.050 |  | 0.90 (0.62-1.28) | 0.548 |
| Elevated CA19-9 | 1.13 (0.64-2.00) | 0.683 |  | 1.22 (0.67-2.21) | 0.517 |  | 1.31 (0.72-2.38) | 0.378 |  | 1.92 (0.83-4.48) | 0.130 |
| RFS |  |  |  |  |  |  |  |  |  |  |  |
| Total | 1.10 (0.92-1.32) | 0.286 |  | 1.19 (0.99-1.43) | 0.069 |  | 1.09 (0.89-1.33) | 0.424 |  | 1.27 (0.98-1.65) | 0.072 |
| Normal CA19-9 | 1.04 (0.85-1.26) | 0.715 |  | 1.11 (0.90-1.35) | 0.326 |  | 1.09 (0.88-1.33) | 0.433 |  | 1.26 (0.97-1.64) | 0.082 |
| Elevated CA19-9 | 1.41 (0.91-2.20) | 0.126 |  | 1.60 (1.01-2.55) | 0.048 |  | 1.80 (1.11-2.90) | 0.016 |  | 1.81 (0.95-3.48) | 0.073 |

Note: CA 19-9, carbohydrate antigen 19-9; CI, confidence interval; HR, hazard ratio; OS, overall survival; RFS, recurrence-free survival.

Model 1 was unadjusted. Model 2 was adjusted for sex (female vs. male), age. Model 3 was adjusted for sex (female vs. male), age, primary site (rectum vs. colon), surgical approach (open resection vs. laparoscopic resection), pathology stage (III→II), lymph node yield (≥12 vs. <12), tumor differentiation (poor-undifferentiated vs. moderate vs. well), mucinous (colloid) type (yes vs. no), lymphovascular invasion / perineural invasion (yes vs. no), adjuvant chemotherapy (yes vs. no). Model 4 was adjusted for sex (female vs. male), age, primary site (rectum vs. colon), surgical approach (open resection vs. laparoscopic resection), pathology stage (III→II), lymph node yield (≥12 vs. <12), tumor differentiation (poor-undifferentiated vs. moderate vs. well), mucinous (colloid) type (yes vs. no), lymphovascular invasion / perineural invasion (yes vs. no), adjuvant chemotherapy (yes vs. no), microsatellite instability (yes vs. no).
